# The Gap Is Getting Closer: Trends in Absolute and Relative Black-White HIV Mortality Disparities in the USA (1999–2023)

**DOI:** 10.1007/s40615-025-02299-8

**Published:** 2025-02-12

**Authors:** Ahmed Azzam, Heba Khaled

**Affiliations:** 1 Department of Microbiology and Immunology, Faculty of Pharmacy, Helwan University, Cairo, Egypt; 2 Department of Biochemistry, Faculty of Pharmacy, Cairo University, Cairo, Egypt

**Keywords:** HIV mortality disparities, Black-White disparities, Absolute and relative gaps, Joinpoint analysis, Racial health inequalities

## Abstract

**Background:**

This study analyzes trends in HIV-related mortality disparities between Black and White populations in the USA from 1999 to 2023.

**Methods:**

CDC WONDER data were utilized to assess absolute disparities, measured as the age-adjusted mortality rate difference, and relative disparities, measured as the age-adjusted mortality rate ratio, in Black-White HIV mortality. Trends were analyzed using Joinpoint regression, with the average annual percentage change (AAPC) serving as a summary measure for the entire period and the annual percentage change (APC) used to evaluate the magnitude of change within specific intervals.

**Results:**

The absolute disparity showed a consistent decline, with an AAPC of − 6.46% (*p* < 0.001). Similarly, the relative disparity significantly decreased, with an AAPC of − 1.10% (*p* < 0.001). A detailed analysis of relative disparity revealed a notable inflection point in 2007, marked by an initial increase from 1999 to 2007 (APC 0.96%, *p* = 0.002), followed by a decline from 2007 to 2023 (APC − 2.12%, *p* < 0.001). The findings for both Black-White females and males consistently demonstrated reductions in both relative and absolute disparities, aligning with the overall trend. Among age categories, the 45–54 age group showed the greatest improvement in absolute (− 8.11%) and relative (− 2.65%) disparities, while the 25–34 group showed the least, with a slight increase in relative disparity (+ 0.23%, *p* = 0.66).

**Conclusion:**

This study shows significant progress in reducing absolute Black-White HIV mortality disparities, while the reduction in relative inequalities is more modest, emphasizing the need for continued and strengthened public health efforts.

## Introduction

Despite advancements in HIV treatment and prevention, HIV-related disparities remain a critical public health challenge, disproportionately impacting Black populations in the USA [1]. In 2020, Black individuals were 7.8 times more likely to be diagnosed with HIV compared to White individuals, illustrating the enduring impact of structural inequities [1]. Beyond higher diagnosis rates, Black individuals also face lower access to HIV pre-exposure prophylaxis (PrEP) and elevated HIV/AIDS-related mortality compared to White populations [2, 3].

Barriers such as limited healthcare access, socioeconomic inequality, structural racism, and gaps in public health infrastructure significantly contribute to these disparities [4, 5]. Addressing these inequities is critical to improving health outcomes and achieving equity, as emphasized by the National HIV/AIDS Strategy (2022–2025), which prioritizes reducing disparities in HIV-related health outcomes for disproportionately impacted populations [6].

Over the past two decades, initiatives like the Minority AIDS Initiative (MAI), the Ryan White CARE Act reauthorization, the CDC’s Expanded HIV Testing Initiative, and grassroots organizations like SisterLove have made meaningful strides in improving access to testing, treatment, and health equity [7–10]. However, the lack of comprehensive evaluation of these efforts highlights the need to analyze long-term trends of HIV-related disparities. To fill this gap, our study evaluates progress in narrowing the Black-White HIV mortality gap from 1999 to 2023 by examining trends in both absolute and relative disparities. These findings aim to illuminate the effectiveness of past interventions and highlight the continued need for sustained, targeted strategies to address persistent health inequities.

## Methods

### Study Design and Data Source

Data was sourced from the CDC WONDER (Wide-ranging Online Data for Epidemiologic Research) Multiple Cause of Death database, which provides county-level mortality and population statistics derived from US death certificates [11]. These certificates record one primary cause of death, along with up to 20 additional causes, and include relevant demographic information. This resource enabled us to analyze the number of deaths, crude death rates, and age-adjusted death rates. In this study, we examined age-adjusted HIV mortality rates per 100,000 population for Black and White individuals using ICD-10 codes B20-B24.

### Employed Metrics for HIV-Related Mortality Disparities

We used two measures to assess disparities in HIV-related mortality between Black and White populations: the age-adjusted mortality rate difference and the age-adjusted mortality rate ratio. The age-adjusted mortality rate difference captures absolute disparity by calculating the difference between the age-adjusted HIV-related mortality rates of the two groups. This measure quantifies the absolute magnitude of the mortality gap, indicating how many more deaths per 100,000 occur in the Black population compared to the White population after adjusting for differences in age distribution. A positive value signifies higher mortality among Black individuals, while a negative value indicates higher mortality among White individuals.

Age-Adjusted Mortality Rate Difference or Absolute Disparity = Age Adjusted Mortality Rate for Blacks − Age-Adjusted Mortality Rate for Whites.

The age-adjusted mortality rate ratio reflects the relative disparity in HIV-related mortality rates between Black and White populations. It indicates the proportional difference in mortality risk between these groups. A ratio greater than 1.0 signifies a higher HIV-related mortality rate in the Black population compared to the White population, while a ratio below 1.0 indicates the opposite. By accounting for differences in age distribution, this measure illustrates how much more (or less) likely Black individuals are to die from HIV compared to White individuals on a relative scale.

Age-Adjusted Mortality Rate Ratio or Relative disparity = Age-Adjusted Mortality Rate for Blacks/Age-Adjusted Mortality Rate for Whites.

In this study, age-adjusted rates were employed instead of crude rates to analyze trends in Black-White HIV-related mortality disparities. The use of age-adjusted rates is critical as they account for variations in age distribution across populations, thereby enabling more accurate and equitable comparisons. This methodological approach is particularly important given the significant role of age as a determinant of mortality risk [12, 13].

Throughout this article, “relative disparity” denotes the age-adjusted mortality rate ratio, while “absolute disparity” represents the age-adjusted mortality rate difference.

### Study Definitions

This study examines two racial groups: Blacks or African Americans (referred to as “Blacks” in this article), defined as individuals with origins in any of the Black racial groups of Africa [14], and Whites, defined as individuals with origins in any of the original peoples of Europe, the Middle East, or North Africa [14]. HIV-related mortality disparities between these racial groups were further analyzed by stratifying data based on Hispanic or Latino ethnicity, which includes individuals of Cuban, Mexican, Puerto Rican, South or Central American, or other Spanish culture or origin, regardless of race [14].

The International Classification of Diseases, Tenth Revision (ICD-10), is a globally standardized system for coding diseases, injuries, and causes of death. Published by the World Health Organization (WHO), ICD-10 provides a consistent framework for classifying and documenting health conditions, enabling a better understanding and analysis of human mortality and morbidity worldwide [15]. Among these codes, B20-B24 classify HIV-related conditions contributing to mortality [16]. These include deaths from opportunistic infections (B20), HIV-associated cancers (B21), specific HIV-related diseases such as encephalopathy (B22), other conditions like acute infection syndrome and lymphadenopathy (B23), and cases where the exact HIV-related complication is unspecified (B24).

Joinpoints are points in a time-series dataset where the trend changes, dividing the data into distinct segments. In Joinpoint Trend Analysis, these points indicate transitions between segments, each with a unique rate of change. The changes at these joinpoints may or may not be statistically significant, but they help identify variations in the trend over time, such as periods of acceleration, deceleration, or stability.

### Study Objectives

The primary objective of this study was to evaluate trends in the Black-White HIV mortality disparity from 1999 to 2023, using two key measures: absolute disparity and relative disparity. The analysis was stratified by sex (male and female), age group (25–34, 35–44, 45–54, 55–64, 65–74, and 75–84 years), and Hispanic ethnicity. However, due to the limited number of mortality cases among Hispanic Black individuals (927 cases), a rigorous statistical analysis for this group was not feasible. As a result, within the Hispanic subgroup, the study focused on comparisons between non-Hispanic Blacks and non-Hispanic Whites, as well as between non-Hispanic Blacks and Hispanic Whites.

### Statistical Analysis

We utilized Joinpoint Trend Analysis Software (Version 5.2.0) [17]. This software is particularly useful for identifying significant changes over time by segmenting the data into distinct periods with varying annual percent changes (APCs), joined at “joinpoints.” The analysis begins with the simplest model, assuming no joinpoints, and iteratively adds joinpoints, testing whether each addition significantly improves the model fit. This process continues up to a maximum of four joinpoints, generating up to five potential models. Model selection is guided by the weighted Bayesian information criterion (WBIC), which balances models fit against complexity, with a lower WBIC value indicating a better model. The significance of changes in trends was assessed using the Monte Carlo Permutation Method, ensuring robust statistical validation. We calculated APCs for each segment to summarize the trend between joinpoints for each factor, while the annual average percent change (AAPC) provided an overall measure of the trend across the entire study period. Confidence intervals were computed for both APCs and AAPCs to quantify uncertainty, allowing for the assessment of the precision of the estimated trends. A *p*-value below 0.05 indicates statistical significance.

## Results

Between 1999 and 2023, there were 225,430 HIV-related deaths in total. Of these, 221,949 (98.46%) occurred within the combined Black and White populations. Among them, 122,759 deaths were in the Black population, corresponding to an age-adjusted mortality rate of 12.45 per 100,000 (95% CI, 12.36–12.53). In comparison, the White population experienced 99,190 HIV-related deaths, with an age-adjusted mortality rate of 1.59 per 100,000 (95% CI, 1.58–1.60). During this period, the overall relative disparity was 7.83, and the absolute disparity was 10.86 per 100,000, as detailed in Table 1.

**Table 1:** Absolute disparity (per 100,000) and relative disparity in HIV-related mortality between Black and White populations, 1999–2023

Variable	Age-adjusted mortality rate per 100,000		Absolute disparity Per 100,000	Relative disparity
	Black or African American	White		
**Total**	12.45	1.59	10.86	7.83
Males	17.99	2.62	15.37	6.87
Females	8.34	0.59	7.75	14.14

Regarding sex, the age-adjusted mortality rate for Black males was 17.99 per 100,000, significantly higher than the 2.62 per 100,000 observed for White males. Among females, the age-adjusted mortality rate was 8.34 per 100,000 for Black females, compared to 0.59 per 100,000 for White females. Males had higher absolute HIV-related death rates, with an absolute disparity of 15.37 per 100,000, while females showed a much greater relative disparity, with a relative disparity of 14.14.

### Trends in the Black-White HIV Mortality Gap, 1999 to 2023

**Fig. 1:**
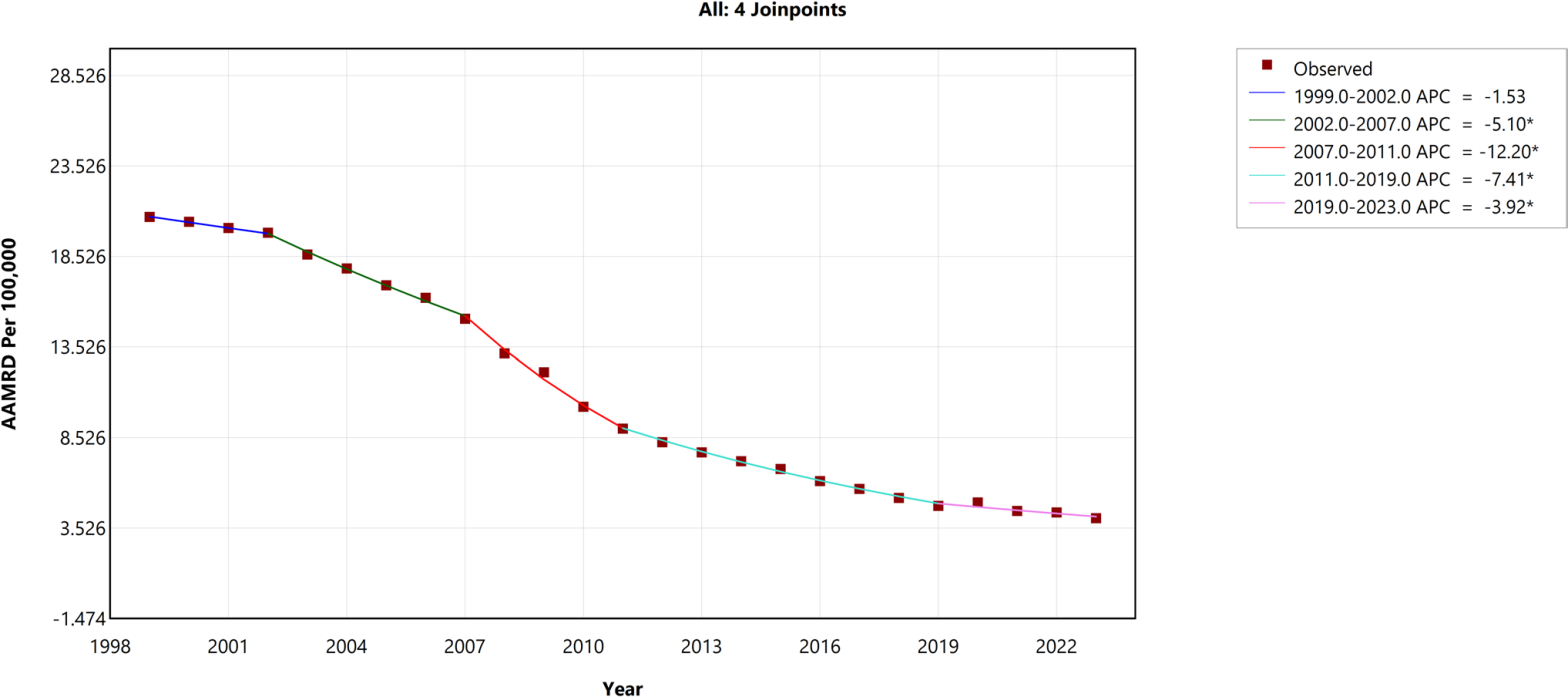
Trends in absolute disparity in the Black-White HIV mortality gap (1999–2023). The *Y*-axis represents the age-adjusted mortality rate difference (AAMRD), denoting absolute disparity, while the *X*-axis represents the years of the study. An asterisk (*) indicates statistical significance where *p*-value is less than 0.05

The trend analysis of the Black-White HIV mortality gap, as measured by the absolute disparity, revealed an AAPC of − 6.46% (95% CI − 6.63 to − 6.29, *p* < 0.001). The segmented trend analysis identified distinct phases across five time periods within the study timeframe, as shown in Fig. 1. In the first segment (1999–2002), the APC was − 1.53% (95% CI − 2.80 to 0.45, *p* = 0.1), indicating a non-significant decline. However, in the second segment (2002–2007), the decline intensified significantly, with an APC of − 5.10% (95% CI − 6.16 to − 4.49, *p* < 0.001). The third segment (2007–2011) saw an even more pronounced acceleration in the rate of decline, with an APC of − 12.20% (95% CI − 13.34 to − 11.23, *p* < 0.001). During the fourth segment (2011–2019), the trend continued downward with an APC of − 7.41% (95% CI − 8.11 to − 6.88, *p* < 0.001). In the final segment (2019–2023), although the rate of decline slowed, it remained statistically significant with an APC of − 3.92% (95% CI − 5.32 to − 1.0, *p* = 0.021).

**Fig. 2:**
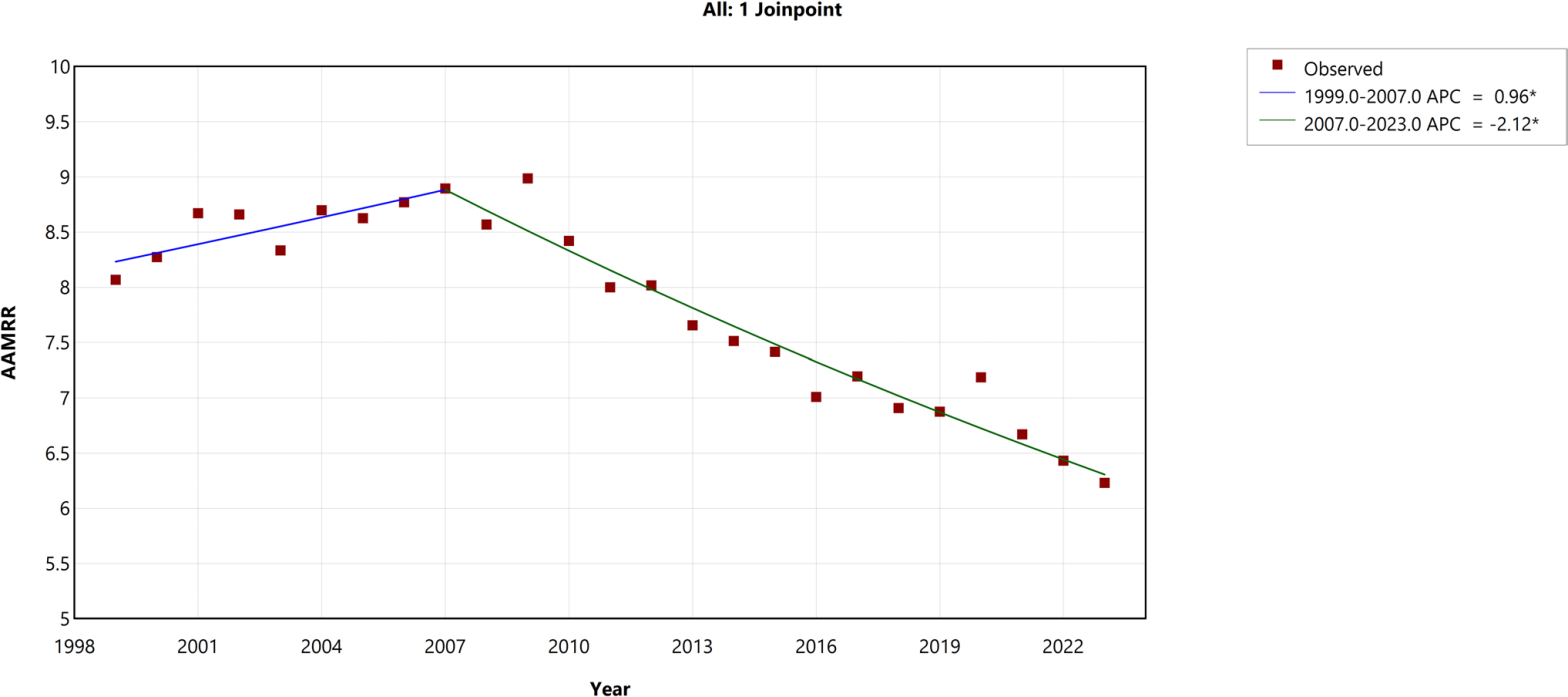
Trends in relative disparity in the Black-White HIV mortality gap (1999–2023). The *Y*-axis represents the age-adjusted mortality rate ratio (AAMRR) denoting realtive disparity, while the *X*-axis represents the years of the study. An asterisk (*) indicates statistical significance where *p*-value is less than 0.05

**Fig. 3:**
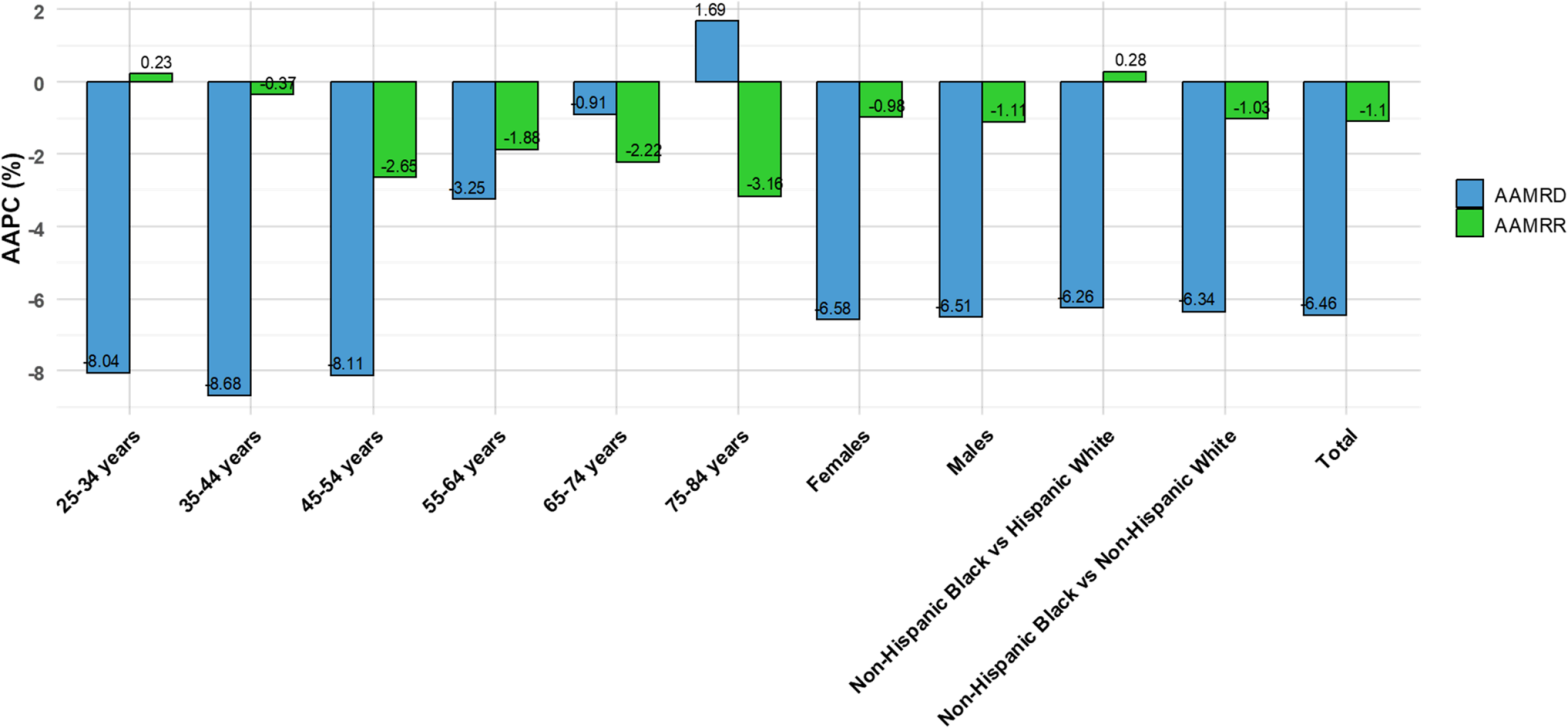
Average annual percentage change (AAPC) in absolute disparity and relative disparity for HIV-related mortality between Black and White populations, stratified by sex, age group, and Hispanic ethnicity (1999–2023). The age-adjusted mortality rate ratio (AAMRR) denotes relative disparity, while the age-adjusted mortality rate difference (AAMRD) denotes absolute disparity

Similarly, the relative disparity demonstrated an AAPC of − 1.10% (95% CI − 1.30 to − 0.94, *p* < 0.001). A more detailed segmented analysis revealed a significant increase in the first segment (1999–2007), with an APC of 0.96% (95% CI 0.34 to 1.78, *p* = 0.0024), followed by a significant decline in the second segment (2007–2023), where the APC was − 2.12% (95% CI − 2.49 to − 1.82, *p* < 0.001), as shown in Fig. 2. The AAPC for absolute and relative disparities for HIV-related mortality between Black-White populations and subgroups by sex, age groups, and Hispanic ethnicity is presented in Table 2 and Fig. 3.

**Table 2:** Trends in HIV-related mortality disparities (1999–2023), stratified by sex, age group, and Hispanic ethnicity

Variable	Absolute disparity		Relative disparity	
	AAPC (95% CI)	*p*-value	AAPC (95% CI)	*p*-value
Total	− 6.46 (− 6.63, − 6.29)	< 0.001	− 1.10 (− 1.30, − 0.94)	< 0.001
Sex				
Black vs. Whites Males	− 6.51 (− 6.70, − 6.33)	< 0.001	− 1.11 (− 1.27, − 0.95)	< 0.001
Black vs. Whites Females	− 6.58 (− 6.85, − 6.32)	< 0.001	− 0.98 (− 1.56, − 0.49)	0.001
Age categories				
25–34 years	− 8.04 (− 9.02, − 7.36)	< 0.001	0.23 (− 0.57, 0.79)	0.66
35–44 years	− 8.68 (− 9.18, − 8.23)	< 0.001	− 0.37 (− 0.98, − 0.01)	0.046
45–54 years	− 8.11 (− 8.54, − 7.80)	< 0.001	− 2.65 (− 3.03, − 2.35)	< 0.001
55–64 years	− 3.25 (− 3.63, − 2.82)	< 0.001	− 1.88 (− 2.21, − 1.47)	< 0.001
65–74 years	− 0.91 (− 1.32, − 0.46)	< 0.001	− 2.22 (− 2.63, − 1.76)	< 0.001
75–84 years	1.69 (0.88, 2.70)	< 0.001	− 3.16 (− 4.09, − 1.88)	< 0.001
Non-Hispanic Black vs Non-Hispanic White	− 6.34(− 6.50, − 6.18)	< 0.001	− 1.03 (− 1.29, − 0.81)	< 0.001
Non-Hispanic Black vs Hispanic White	− 6.26 (− 6.53, − 6.04)	< 0.001	0.28(− 0.23, 0.70)	0.21

*AAPC* average annual percentage change, *CI* confidence interval

### Trends in the Black-White HIV Mortality Gap by Sex, 1999 to 2023

**Fig. 4:**
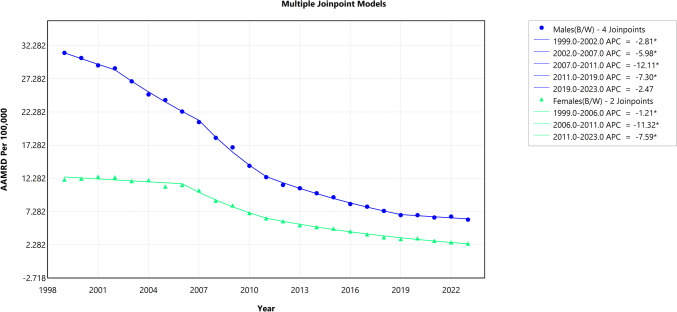
Trends in absolute disparity difference by sex for Black-White HIV mortality gap (1999–2023). The *Y*-axis represents the age-adjusted mortality rate difference (AAMRD), denoting absolute disparity, while the *X*-axis represents the years of the study. An asterisk (*) indicates statistical significance where *p*-value is less than 0.05

**Fig. 5:**
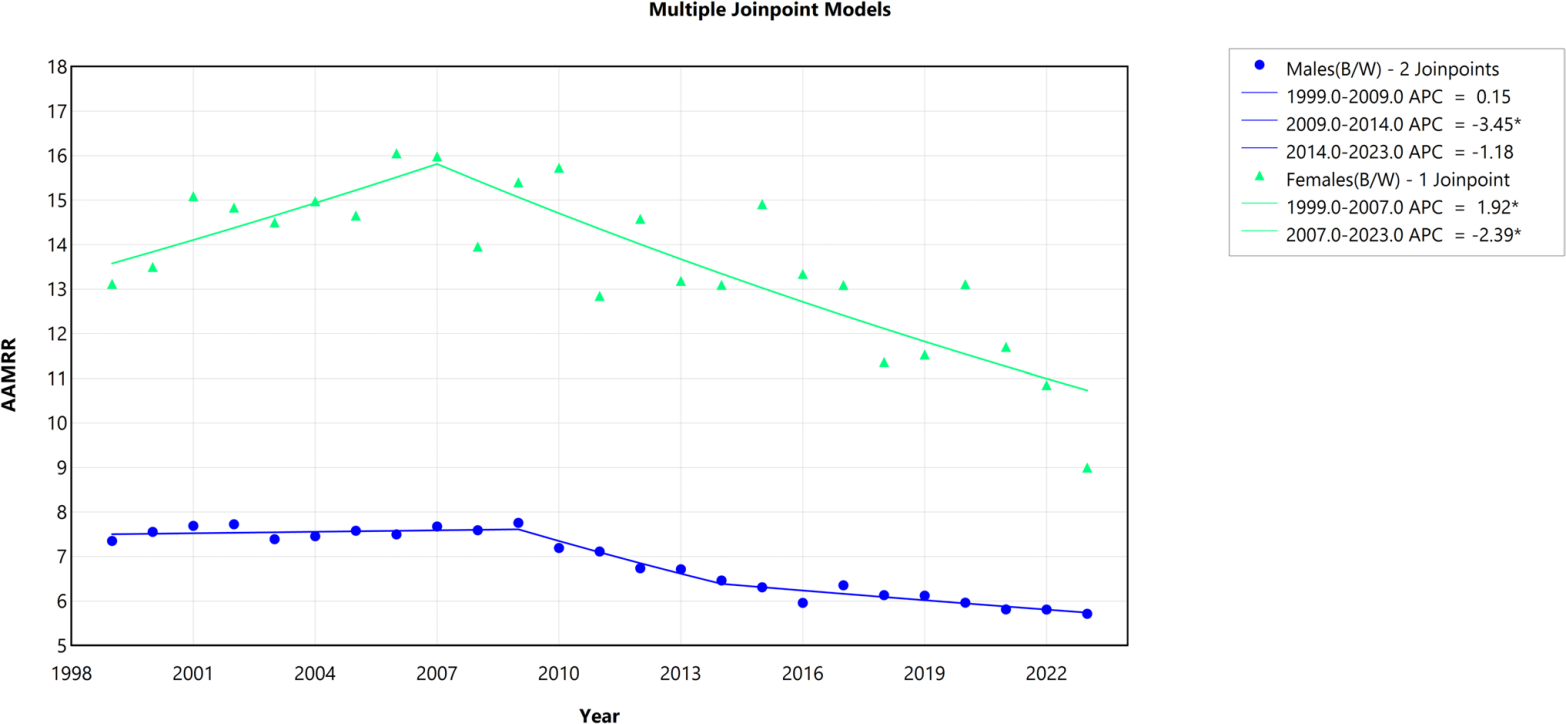
Trends in relative disparity by sex for Black-White HIV mortality (1999–2023). The *Y*-axis represents the age-adjusted mortality rate ratio (AAMRR), denoting relative disparity, while the *X*-axis represents the years of the study. An asterisk (*) indicates statistical significance where *p-*value is less than 0.05

The trend analysis of absolute disparity showed that the AAPC for Black-White males was − 6.51% (95% CI − 6.70 to − 6.33, *p* < 0.001), while for Black-White females, it was − 6.58% (95% CI − 6.85 to − 6.32, *p* < 0.001). In contrast, the trend analysis of the Black-White HIV mortality gap, measured using relative disparity, indicated an AAPC of − 1.11% for Black-White males (95% CI − 1.27 to − 0.95, *p* < 0.001) and − 0.98% for Black-White females (95% CI − 1.56 to − 0.49, *p* = 0.001). Detailed trend segments of the Black-White HIV mortality in absolute disparity and the relative disparity gap by sex are shown in Figs. 4 and 5, respectively.

### Trends in the Black-White HIV Mortality Gap by Age Categories, 1999 to 2023

**Fig. 6:**
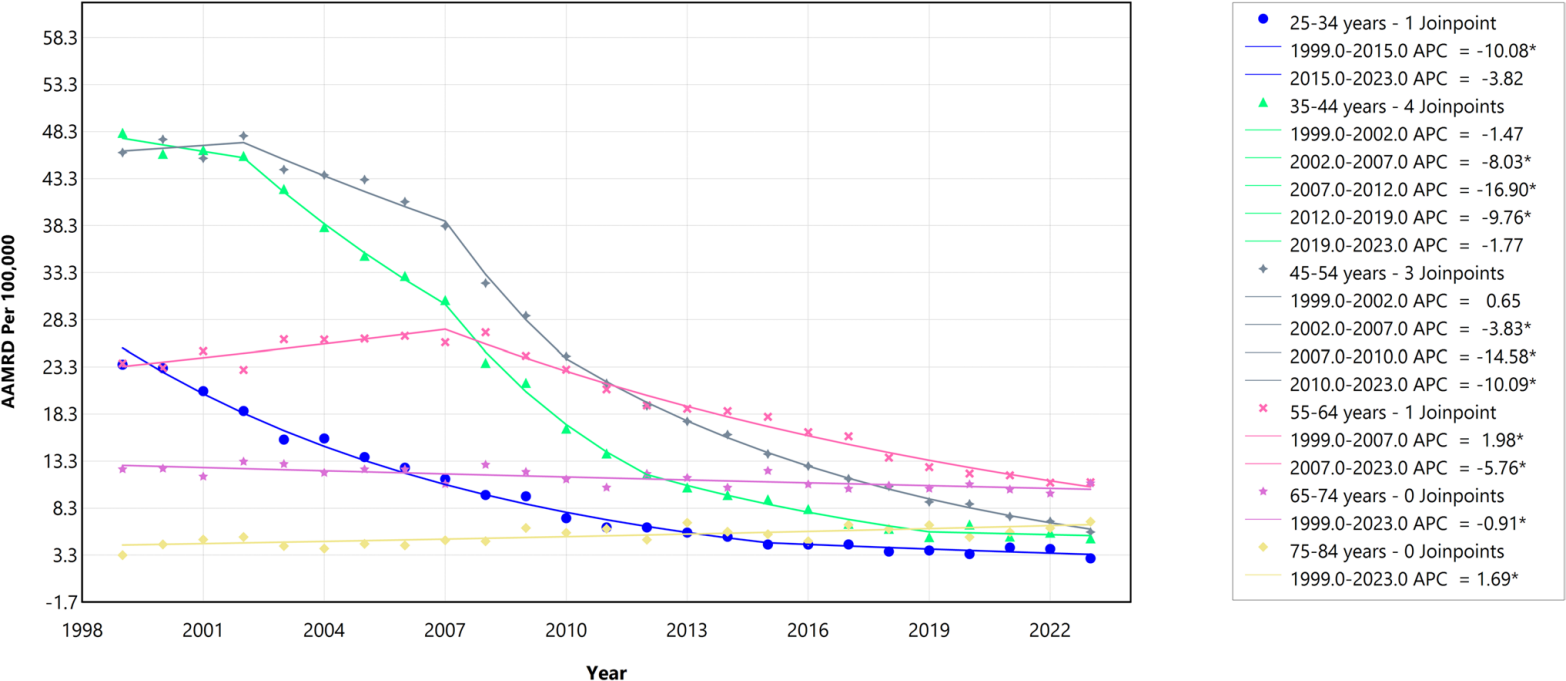
Trends in absolute disparity in the Black-White HIV mortality by age categories (1999–2023). The *Y*-axis represents the age-adjusted mortality rate difference (AAMRD) denoting absolute disparity, while the *X*-axis represents the years of the study. An asterisk (*) indicates statistical significance where *p*-value is less than 0.05

Regarding age cohorts, the 35–44 years cohort experienced the largest decrease in the absolute disparity, with an AAPC of − 8.68% (95% CI − 9.18 to − 8.23, *p* < 0.001), followed by the 45–54 years cohort, which had an AAPC of − 8.11% (95% CI − 8.54 to − 7.80, *p* < 0.001). The 55–64 years cohort showed a more moderate decline, with an AAPC of − 3.25% (95% CI − 3.63 to − 2.82, *p* < 0.001), while the 65–74 years cohort experienced a smaller decrease, with an AAPC of − 0.91% (95% CI − 1.32 to − 0.46, *p* < 0.001). In contrast, the 75–84 years cohort showed an increase in the gap, with an AAPC of 1.69% (95% CI 0.88 to 2.70, *p* < 0.001), as shown in Fig. 6.

Trend analysis of the relative disparity revealed that the 45–54 years cohort experienced the largest decrease, with an AAPC of − 2.65% (95% CI − 3.03 to − 2.35, *p* < 0.001), followed by the 75–84 years cohort, which had an AAPC of − 3.16% (95% CI − 4.09 to − 1.88, *p* < 0.001). The 55–64 years cohort showed a significant decline, with an AAPC of − 1.88% (95% CI − 2.21 to − 1.47, *p* < 0.001), while the 65–74 years cohort had a similar decrease, with an AAPC of − 2.22% (95% CI − 2.63 to − 1.76, *p* < 0.001). The 35–44 years cohort saw a more moderate decline, with an AAPC of − 0.37% (95% CI − 0.98 to − 0.01, *p* = 0.047). In contrast, the 25–34 years cohort showed no significant change, with an AAPC of 0.23% (95% CI − 0.57 to 0.79, *p* = 0.657).

### Trends in the Black-White HIV Mortality Gap by Hispanic Origin, 1999 to 2023

**Fig. 7:**
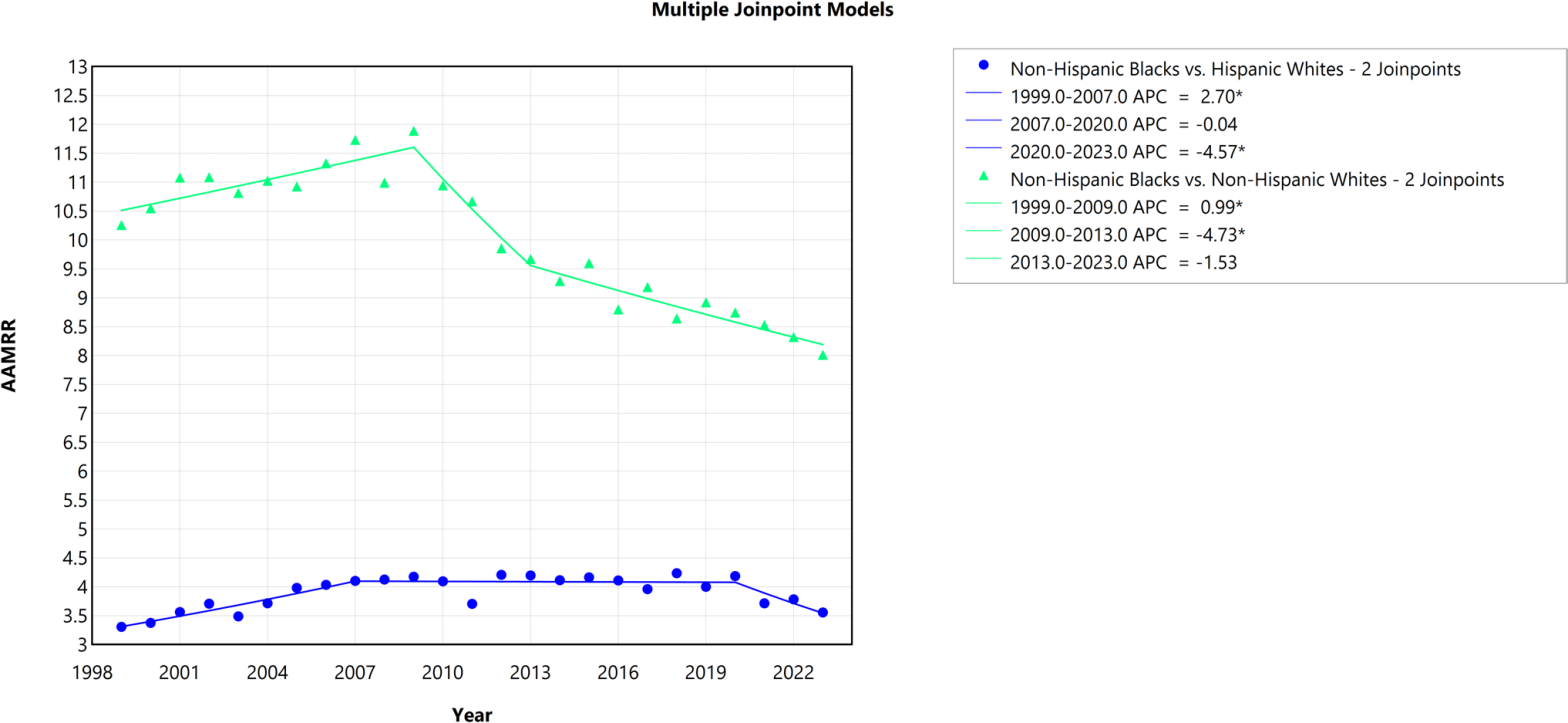
Trends in relative disparity in the Black-White HIV mortality by Hispanic ethincity (1999–2023). The *Y*-axis represents the age-adjusted mortality rate ratio (AAMRR) denoting realtive disparity, while the *X*-axis represents the years of the study. An asterisk (*) indicates statistical significance where *p*-value is less than 0.05

The trend analysis of absolute disparity between non-Hispanic Black and non-Hispanic White populations shows a significant decline, with an AAPC of − 6.34% (95% CI − 6.50 to − 6.18, *p* < 0.001). Detailed analysis revealed a consistent decrease throughout the study period, with no reversals observed. Similarly, the relative disparity between these groups experienced a more modest decline, with an AAPC of − 1.03% (95% CI − 1.29 to − 0.81, *p* < 0.001). A closer examination of the segmented trends reveals distinct phases. From 1999 to 2009, there was a slight but significant increase, with an APC of 0.99% (95% CI 0.38 to 1.66, *p* = 0.03). Between 2009 and 2013, the disparity decreased significantly, with an APC of − 4.73% (95% CI − 7.00 to − 0.01, *p* = 0.05). From 2013 to 2023, the decline slowed, showing stabilization, with an APC of − 1.53% (95% CI − 2.38 to 1.16, *p* = 0.13), as shown in Fig. 7.

For the absolute disparity between non-Hispanic Black and Hispanic White populations over the same period, there was also a significant decline, with an AAPC of − 6.26% (95% CI − 6.53 to − 6.04, *p* < 0.001). Detailed analysis revealed a consistent decrease throughout the study period, with no reversals observed. In contrast, the relative disparity between these groups showed a small, non-significant increase, with an AAPC of 0.28% (95% CI − 0.23 to 0.70, *p* = 0.21). Detailed analysis revealed three distinct phases: from 1999 to 2007, there was a significant increase, with an APC of 2.70% (95% CI 0.68 to 5.77, *p* = 0.03); between 2007 and 2020, the trend plateaued, with an APC of − 0.04% (95% CI − 0.58 to 4.57, *p* = 0.93); however, this trend reversed from 2020 to 2023, with a significant decrease observed, reflected by an APC of − 4.57% (95% CI − 11.63 to − 0.45, *p* = 0.006), as shown in Fig. 7.

## Discussion

Our study examines trends in absolute and relative disparities in HIV-related mortality between Black and White populations in the USA from 1999 to 2023. The findings reveal significant progress in reducing absolute disparities, reflecting improvements in overall mortality rates. However, while the relative disparities between the two groups have also shown a statistically significant decrease, the reduction remains modest. This highlights the ongoing need for sustained efforts to achieve health equity by addressing relative disparities more effectively. Tackling underlying systemic factors such as socioeconomic inequality, limited healthcare access, stigma, and the need for tailored community health interventions is critical to closing these gaps and ensuring equitable health outcomes.

The HIV epidemic in the USA has evolved significantly since it was first identified in the early 1980s [18]. While life expectancy and quality of life for individuals living with HIV have improved [19–21], significant disparities persist, particularly among racial and ethnic minorities [1]. Black individuals in the USA face disproportionately high rates of new HIV diagnoses and HIV-related mortality [3, 22]. This study highlights significant disparities, with an overall relative disparity of 7.83 and an absolute disparity of 10.86 per 100,000. The disproportionately high HIV-related mortality rates among Black individuals in the USA result from a combination of systemic barriers to timely diagnosis and treatment, limited access to quality healthcare, socioeconomic inequalities that exacerbate health disparities, and the impact of stigma and discrimination, which hinder engagement with care and adherence to life-saving treatments [4, 23–26].

Our study also revealed notable gender differences in HIV-related mortality. Black males exhibit higher absolute HIV-related mortality rates, with an absolute disparity of 15.37 per 100,000 compared to 7.75 per 100,000 for Black and White females. This disparity is driven by the higher baseline prevalence of HIV in men compared to women, coupled with disproportionately elevated HIV-related mortality rates among Black men relative to their White counterparts. On the other hand, the relative disparity between Black and White women is significantly larger—more than twice as high at 14.14 compared to 6.87 for Black and White men. This suggests that, while systemic barriers hindering access to HIV care exist among Black males compared to White males, these barriers are even more pronounced for Black females compared to White females. For example, Black women often report more negative experiences within the healthcare system; 52% of Black women aged 18 to 49 have had to advocate for themselves to receive proper care, compared to 29% of Black men in the same age group [27]. Additionally, Black women experienced the highest poverty rates compared to both White women, White men, and Black men, further limiting their access to adequate healthcare resources and intensifying existing disparities [28].

This study demonstrates a significant reduction in both absolute and relative HIV-related mortality disparities, driven by advancements in treatment and public health initiatives. The trend analysis for absolute disparities revealed a consistent decline, with an AAPC of − 6.46% (*p* < 0.001) throughout the study period. This decline was particularly pronounced between 2007 and 2011, where the APC reached − 12.20% (*p* < 0.001). This acceleration coincides with the introduction of highly effective antiretroviral therapies before this period, such as tipranavir (2005), a combination of efavirenz, emtricitabine, and tenofovir disoproxil fumarate (2006), darunavir (2006), maraviroc (2007), and raltegravir (2007) [29].

On the other hand, the relative disparity in HIV-related mortality between Black-White individuals declined modestly, decreasing from 8.06 to 6.2 (AAPC − 1.10%, *p* < 0.001). A more detailed analysis revealed an inflection point in 2007, where the relative disparity shifted from an increasing trend to a significant decline. This shift can be attributed to key public health initiatives implemented around this period, which significantly improved early HIV diagnosis, access to antiretroviral therapy, and overall care for underserved populations, particularly within Black communities. These efforts contributed to a faster decline in HIV-related mortality rates among Black individuals compared to White individuals, thereby narrowing the relative disparity over time. A prominent example is the Expanded HIV Testing Initiative implemented by the CDC in 2007 [7]. Published findings from this initiative demonstrated a significant reduction in the prevalence of undiagnosed HIV among Black individuals and facilitated robust linkage to care for the majority of newly diagnosed cases [7]. Black populations, in particular, benefited from improved access to healthcare services [7]. Additionally, the continued support for the Minority AIDS Initiative (MAI) throughout the 2000s and the expansion of the Ryan White HIV/AIDS Program in 2006 played pivotal roles in addressing HIV-related disparities [10, 30].

Our findings are consistent with a previous CDC report that noted both absolute and relative disparities in HIV diagnosis rates between Black-White women decreased [31]. The absolute disparity decreased from 36.9 to 28.3 per 100,000, and the relative disparity saw a reduction as well, with the disparity ratio declining from 1.7 to 1.2. A prior study addressing the trend in Black-White HIV-related mortality disparities in the USA from 1990 to 2009 revealed a notable increase in the Black-White mortality rate ratio [32]. The disparity between the two groups worsened significantly over time, with the rate ratio rising from 4.33 in 1990–1994 to 11.38 in 2005–2009 [32]. While this study provides valuable insights into that period, it has some limitations. For instance, it primarily relies on rate ratios to measure relative disparities, which offer a proportional view but do not capture absolute differences in mortality rates. Additionally, the study analyzes only fixed 5-year increments, which may miss subtle trend shifts within these periods, limiting the ability to detect more granular changes in disparities over time. Furthermore, the study measures HIV-related mortality disparities using crude rates instead of age-adjusted rates, which can distort the true gap.

The 45–54 age group demonstrated the most significant improvement in both absolute and relative HIV-related mortality disparities, with substantial reductions in absolute disparity (AAPC − 8.11%, *p* < 0.001) and relative disparity (AAPC − 2.65%, *p* < 0.001). Several factors likely contributed to this progress, including targeted public health initiatives, increased healthcare engagement, and greater socioeconomic stability. Individuals in this age group often benefit from stable employment and health insurance, which facilitate regular medical check-ups and adherence to HIV treatment. In contrast, the 25–34 age group showed the least progress. While the absolute disparity decreased significantly (− 8.04%, *p* < 0.001), the relative disparity showed a nonsignificant increase (+ 0.23%, *p* = 0.66), indicating no meaningful improvement in relative disparity. The plateauing of relative disparities among the 25–34 age group is likely attributed to socioeconomic instability, delayed healthcare engagement, persistent stigma, and fewer targeted interventions compared to older age groups. To address this issue, targeted efforts should prioritize reducing both absolute and relative disparities in HIV-related mortality within this younger population.

## Strength and Limitations

This study offers a comprehensive analysis of both absolute and relative disparities in Black-White HIV-related mortality over a substantial period (1999–2023) using CDC WONDER data. By applying Joinpoint regression analysis, the study effectively identifies significant trends and shifts in mortality disparities, providing valuable insights into the effectiveness of public health interventions. A key strength is the focus on age-adjusted mortality rates, which allows for a more accurate comparison of mortality trends across different demographic groups, minimizing the effects of age distribution differences between populations. Additionally, the study addresses both absolute and relative disparities, capturing a nuanced understanding of progress in reducing overall mortality while addressing remaining proportional inequalities. The stratification of Black-White disparities by sex, ethnicity, and age groups further enhances the study’s value, providing a detailed analysis of which populations have benefited most from interventions. Notably, it highlights the slower progress observed among younger age groups, warranting further attention. The study also addresses variations by Hispanic ethnicity. However, the study has some limitations. It relies on aggregate mortality data, which may mask important within-group variations, such as socioeconomic status, geographic location, or access to healthcare, limiting the ability to pinpoint specific drivers of disparities.

## Conclusion

This study shows significant progress in reducing absolute Black-White HIV mortality disparities, while the reduction in relative inequalities was more modest. The non-significant increase in relative disparities in the 25–34 age group underscores the need for continued efforts to address these inequalities.

## Data Availability

The data were retrieved from CDC WONDER (Multiple Causes of Death) (https://wonder.cdc.gov) and are publicly accessible per CDC terms.
